# Supplementary material for: Collaborative intelligence in AI: Evaluating the performance of a council of AIs on the USMLE
Source: PLOS Digit Health. 2025 Oct 9;4(10):e0000787. doi: 10.1371/journal.pdig.0000787 (PMC12510544; doi:10.1371/journal.pdig.0000787)
Supplement: S2 File — (ZIP) [file pdig.0000787.s002.zip › S2 Data Raw data files containing unprocessed question inputs and LLM outputs including transcripts of deliberations/STEP3_Question-82-1_Type-MC-NJ.docx]

**Facilitator** **[Round 1]:** A 32-year-old woman, gravida 4, para 4, comes to the office 1 week after an uncomplicated vaginal delivery of a 3020-g (6-lb 11-oz) term female newborn. She has been breast-feeding her daughter since birth. The patient says that she cries frequently for no reason, is irritable, and is worried about her infant's long-term health. The patient reports having no appetite. She says that her husband and mother say that she is depressed and think she is anorexic. She has not had auditory hallucinations, confusion, or disorientation. She denies suicidal or homicidal ideation. She has a history of postpartum psychosis following the birth of her first child; she had no similar symptoms after the births of her second and third children. Which of the following factors in this patient's history most strongly indicates a poor prognosis? (A) Anorexia (B) Depressed mood (C) History of psychosis (D) Multiparity

**AI_C:** RESTATING THE QUESTION: This query is about a 32-year-old female who has given birth four times and is presenting with symptoms of depression one week after her most recent childbirth. Additionally, she has a history of postpartum psychosis following her first childbirth. The question pertains to figuring out which factor in her history is indicative of a negative progression or poor prognosis of her condition. PLANNING: Plainly stated, the assumptions include: anorexia can cause a poor prognosis, a depressed mood can result in a poor prognosis, a history of psychosis can lead to a poor prognosis, and multiparity (having multiple births) might result in a poor prognosis. After questioning these assumptions, the idea emerges that her history of psychosis could probably be the strongest indication of a poor prognosis. This is based on the fact that she did not experience similar symptoms after the subsequent childbirths, and the current depressive symptoms might point towards a re-emergence of a psychiatric condition. CLINICAL REASONING: Postpartum depression often presents with symptoms such as irritability, tearfulness, and anxiety, which this patient seems to be exhibiting. However, this condition is typically transient and tends not to have long-term adverse effects. On the other hand, postpartum psychosis, which might have occurred with her first childbirth, is a severe condition that can have a significant impact on a woman’s future mental health. Given the severity of this disorder and the potential for it to recur, a history of psychosis could be seen as a strong indicator of a poor prognosis. CONCLUDING: In summary, while this woman is currently exhibiting depressive symptoms (depressed mood), these would not have as poor a prognosis as a history of psychosis, which is her most severe past psychiatric disorder. In light of this, it appears that her previous episodes of psychosis suggest a higher risk of psychiatric disturbances in the future. RESPONSE: So, the factor in this woman's history that most strongly suggests a poor prognosis is her history of psychosis (C).

**AI_D:** RESTATING THE QUESTION: The question pertains to a 32-year-old woman who has had her fourth child. The woman reports experiencing a change in mood, irritability, lack of appetite, concerns about her newborn's health, and has a previous history of postpartum psychosis during her first childbirth. However, she had no similar issues with her second and third children. The question we're asked to consider is which item from her history suggests the poorest prognosis - her anorexia, her depression, her history of psychosis, or her multiparity, or having had more than one childbirth. PLANNING: Let's understand: "anorexia" refers to a severe lack of appetite or aversion to food; "depression" is a mood disorder manifesting with prolonged sadness, interest loss, and other psychological symptoms; "history of psychosis" refers to previous psychotic episodes, and psychosis is a severe mental disorder causing a disconnection from reality; "multiparity" refers to the state of having given birth more than once. Each factor signifies its potential risk, yet we need to determine which signifies the greatest risk. CLINICAL REASONING: In terms of prognosis, it's critical to consider factors that will persist over time or indicate recurrent problems. While lack of appetite and depressed mood are concerning, these might presumably be transient and improve with appropriate support, therapy, and drugs where applicable. Multiparity, too, is notable but does not contribute directly to her existing symptoms. Research suggests that new mothers are equally likely to experience mental health issues, irrespective of which birth they're on. However, a history of psychosis is particularly significant. Postpartum psychosis is infrequent, but when it transpires, the woman is at elevated risk of recurrence with consequent pregnancies. A history of postpartum psychosis is acknowledged as a significant predictor for recurrence and strongly correlated with bipolar disorder - a lifelong, recurrent disorder requiring lifelong treatment. CONCLUDING: The history of psychosis presents the most significant cause for concern, considering the recurrence potential and its relation to severe mental disorders like bipolar disorder. Therefore, this could have the most significant impact on her prognosis. RESPONSE: The factor in the patient's history that most strongly indicates a poor prognosis is (C) History of psychosis.

**AI_A:** (1) RESTATING THE QUESTION: A mother of four children, who is one week postpartum from the delivery of her latest child, visits the health office. She has been nurturing her infant through breastfeeding and is concerned about her baby's future wellbeing. She experiences unexplained crying spells, mood irritability, and decreased food intake, which has prompted her family members to categorize her as depressed and potentially anorexic. She had experienced postpartum psychosis with her first child in the past, but not with her subsequent two children. She denies experiencing any symptoms indicative of psychosis currently. The aim is to identify which among her signs, symptoms, and history would be a strong indication of a negative(e.g. poor) outcome or prognosis. We have - (A) Anorexia (B) Depressed mood (C) History of psychosis (D) Having multiple children (Multiparity) (2) PLANNING: To determine which of these factors most likely indicates a poor prognosis, we need to consider the specific implications of each factor and what they mean in context of postpartum mental health. The factors are anorexia, depressed mood, history of psychosis, and multiparity. It would seem intuitive that a severe condition like a history of postpartum psychosis could indicate a worse prognosis than anorexia or depressive mood, as the aftermath may continue to affect the person. But before coming to a conclusion, we need to evaluate each choice carefully and generate some hypotheses. (3) CLINICAL REASONING: 'Anorexia' and 'depressed mood' are common symptoms in depressive disorders, particularly postpartum depression, and while they definitely need attention, they do not necessarily indicate a worse prognosis. Depression is treatable, and a patient's prognosis can be very good with appropriate therapy and medications. 'Multiparity' could be associated with higher risk of certain physical complications, but there's no clear-cut correlation between the number of pregnancies and mental health prognosis. A 'history of psychosis', particularly postpartum psychosis, on the other hand, is a strong risk factor for recurrence in future pregnancies and postpartum periods and it has significant clinical implications. Postpartum psychosis is a rare and severe disorder that represents a psychiatric emergency and can have serious consequences if not managed properly. It typically comprises symptoms such as delusion, hallucination and distorted thinking. (4) CONCLUDING: In this case, we synthesize the understanding of these four factors with what we know about postpartum mental health, it appears that the patient's 'history of psychosis' most strongly indicates a poor prognosis. This implies that the patient's past experience with postpartum psychosis could have long-term effects that influence her mental health prognosis more significantly than the other factors presented. (5) RESPONSE: Hence, for this 32-year-old woman, a week postpartum from her fourth delivery, and exhibiting signs of mood irritability, unexplained crying and decreased appetite, the factor that most strongly indicates a poor prognosis is her past 'History of psychosis' (Choice C).

**AI_B:** RESTATING THE QUESTION: The question is about a 32-year-old woman who has given birth 4 times (gravida 4, para 4). She recently delivered a healthy baby girl without any complications. She's been experiencing symptoms suggestive of a depressive state including frequent crying, irritability, worries about her baby's health, loss of appetite, and perceived feelings of depression and potential anorexia noticed by her family members. There's no evidence of any psychotic symptoms such as hallucinations, confusion, or disorientation, nor imminent harm her or others (suicidal or homicidal ideation). She has a past history of postpartum psychosis that happened only after the birth of her first child but not her second and third children. The question is asking which factor amongst her anorexia, depressed mood, past history of psychosis, and multiparity (giving birth to more than one child), would contribute to a worse prognosis. PLANNING: Initially, we assume that all four options provided could potentially influence her prognosis. We can however, generate an alternate assumption where we consider the woman's past history of postpartum psychosis as being the strongest predictor of a poor prognosis. This is because a history of psychiatric disorders, especially related to childbirth (like postpartum psychosis), puts women at higher risk for future psychiatric issues, independent of other potential contributing factors like diet or mood. CLINICAL REASONING: There is a clear discrepancy in the patient's postnatal history. She has had an episode of postpartum psychosis after her first childbirth but has not had similar symptoms after her second and third child. Given her current symptoms of depression and anorexia, it's clear that she is experiencing a differing manifestation of mental health issues after her fourth childbirth. Her mood symptoms resonate with postpartum depression rather than postpartum psychosis. However, looking back at her past psychiatric history and the current depressive presentation in the absence of psychotic features, one could infer a pattern of reproductive-related mood swings that represents significant emotional instability. This persistent instability despite different symptomatic manifestations indicates a complex mental health matrix, which is not easily resolved and therefore could lay a foundation for a long-lasting or recurrent psychiatric ailment. Histories of psychiatric disorders, especially severe ones like psychosis, creates vulnerability to future mental health issues. Therefore, the woman's history of postpartum psychosis indeed poses a grave concern. CONCLUDING: Based on her clinical history and symptomatology, while her anorexia and depressed mood are causes for concern, they seem to be symptomatic of underlying psychiatric distress rather than independent contributing factors to a poor prognosis. Multiparity, although a significant physiological stressor, does not necessarily predict a poor prognosis, especially in the context of prior pregnancies without psychiatric sequelae. RESPONSE: (C) The factor from the patient's history that most strongly indicates a poor prognosis is her history of postpartum psychosis. Despite not having similar symptoms after her other pregnancies, this severe episode in her history creates a vulnerability to future mental health disturbances and therefore indicates a worse prognosis.

**AI_E:** RESTATING THE QUESTION: The patient, a 32-year-old mother of four, is experiencing depressive symptoms and loss of appetite after her recent childbirth. Despite a previous episode of postpartum psychosis after her first childbirth, her second and third deliveries were uneventful. The aim is to identify which among her current and past conditions—specifically anorexia, a depressed mood, her history of psychosis, or the fact she has had multiple pregnancies—has the strongest implications for a poor prognosis. PLANNING: To address this, we must navigate the assumptions around anorexia, depressed mood, previous psychosis, and multiparity, their possible effect on postpartum mental health, and their associated risks of recurring. Considering all these anomalies, a case could be made for prioritizing the examination of her history of psychosis as it stands out due to its severity and possible likelihood of recurrence in the light of her current depressive symptoms. CLINICAL REASONING: Our evaluation of the patient's potential for a less favorable prognosis needs to take into account both her present condition and her personal psychiatric history. While her current symptoms mirror those typical of postpartum depression or baby blues, her past history of postpartum psychosis is of particular interest. This is because, typically, patients with a prior episode of postpartum psychosis have a 50-62% risk of recurrence with subsequent pregnancies. Furthermore, the general population risk for postpartum psychosis is about 1-2 per 1,000 deliveries, and the risk increases to 100 per 1,000 in women with a history of postpartum psychosis. Although no psychosis symptoms are reported in her current presentation, her depressive mood could potentially progress into a more severe disorder like postpartum psychosis, given her history. CONCLUDING: Keeping this in mind, it could therefore be hypothesized that the factor in her history that most strongly indicates a poor prognosis would most likely be her previous episode of postpartum psychosis. RESPONSE: The correct answer is (C) History of psychosis. Among all factors presented in this patient's history, her past postpartum psychosis after her first childbirth potentially carries the strongest implication for a poor prognosis given the high chances of recurrence of this severe condition. Her present depressive symptoms add to this concern, regardless of her current absence of psychosis symptoms.

**Facilitator** The consensus statement synthesizes the responses of AI_A, AI_B, AI_C, AI_D, and AI_E as follows: The question pertains to a 32-year-old woman who has recently given birth to her fourth child. She is currently experiencing symptoms of depression and anorexia, and has a past history of postpartum psychosis following her first childbirth. The aim is to identify which among her current and past conditions—specifically anorexia, a depressed mood, her history of psychosis, or the fact she has had multiple pregnancies—has the strongest implications for a poor prognosis. In evaluating these factors, it is important to consider their potential long-term impact and likelihood of recurrence. While anorexia and depressed mood are concerning, they are often transient and can improve with appropriate treatment. Multiparity, or having multiple pregnancies, does not directly contribute to her current symptoms and does not necessarily predict a poor prognosis. However, her history of postpartum psychosis is particularly significant. Postpartum psychosis is a severe mental disorder that can have a substantial impact on a woman’s future mental health. Given the severity of this disorder and the potential for it to recur, a history of psychosis could be seen as a strong indicator of a poor prognosis. Despite not having similar symptoms after her other pregnancies, this severe episode in her history creates a vulnerability to future mental health disturbances and therefore indicates a worse prognosis. Therefore, the consensus among the responses is that the factor in this woman's history that most strongly suggests a poor prognosis is her history of psychosis (C). This is due to the severity of postpartum psychosis, its potential for recurrence, and its association with other severe mental disorders. Her current depressive symptoms add to this concern, regardless of her current absence of psychosis symptoms.
